# NiTi_2_, a New Liquid Glass

**DOI:** 10.3390/ma16206681

**Published:** 2023-10-13

**Authors:** Robert F. Tournier, Michael I. Ojovan

**Affiliations:** 1 UPR 3228 Centre National de la Recherche Scientifique, Laboratoire National des Champs Magnétiques Intenses, European Magnetic Field Laboratory, Institut National des Sciences Appliquées de Toulouse, Université Grenoble Alpes, F-31400 Toulouse, France; robert.tournier@lncmi.cnrs.fr; 2 Department of Materials, Imperial College London, London SW7 2AZ, UK

**Keywords:** glass, phase transitions, liquid glass, configurons, molecular dynamic simulations

## Abstract

Many endothermic liquid–liquid transitions, occurring at a temperature T_n+_ above the melting temperature T_m_, are related to previous exothermic transitions, occurring at a temperature T_x_ after glass formation below T_g_, with or without attached crystallization and predicted by the nonclassical homogenous nucleation equation. A new thermodynamic phase composed of broken bonds (configurons), driven by percolation thresholds, varying from ~0.145 to Δε, is formed at T_x,_ with a constant enthalpy up to T_n+_. The liquid fraction Δε is a liquid glass up to T_n+_. The solid phase contains glass and crystals. Molecular dynamics simulations are used to induce, in NiTi_2_, a reversible first-order transition by varying the temperature between 300 and 1000 K under a pressure of 1000 GPa. Cooling to 300 K, without applied pressure, shows the liquid glass presence with Δε = 0.22335 as memory effect and T_n+_ = 2120 K for T_m_ = 1257 K.

## 1. Introduction

The classical homogenous nucleation (CHN) equation [1,2,3,4] cannot be applied above the melting temperature T_m_ because the melt is viewed as being homogenous, excluding the presence of intrinsic nuclei such as superclusters and superatoms, inducing crystallization after undercooling and liquid–liquid transitions above T_m_ [5]. The liquid–liquid transitions cannot be related to a first-order transition occurring below T_m_. The undercooling of liquid elements gradually increases with overheating and reaches an undercooling plateau, which is attributed to heterogenous nucleation from oxide impurities, surface cavities, and growth kinetics dependent with the size instead of homogenous nucleation [6]. Scientists were quasi-unanimous at the end of 20th century, adopting CHN, believing that the glassy state results from liquid freezing instead of homogenous nucleation of phase transition.

The undercooling plateau is defined by the weakest reduced temperature θ = (T − T_m_)/T_m_ where the crystallization occurs for a volume v and a nucleation time t depending on the cooling rate. The product θ^2^ (1 + θ) for vt = 10^−8 ± 1^ m^3^s in the CHN equation is a linear function of α^3^S_m_ where α is proportional to the surface energy of the growth nuclei and S_m_ is the entropy of melting of 38 elements. In fact, this law, deduced from the CHN equation, excludes the heterogenous nucleation and promotes the homogenous nucleation and the presence of intrinsic nuclei in melts [7].

The nonclassical homogenous nucleation (NCHN) equation includes the possible presence of homogeneous nuclei, introducing in the classical equation their contribution to the Gibbs free energy [7]. The homogenous nucleation temperatures (T_n−_) are defined by equations of two liquid states, Liquid 1 and Liquid 2 [5,8,9,10,11]. The formation of glassy phases occurs by percolation of bonds at T_g_ during the first cooling [12,13,14]. After quenching the melt below T_g_, the enthalpy of formation of all bonds can relax. There are two homogeneous nucleation temperatures in Liquid 2. The highest is (T_n−_ = T_g_); the lowest is the temperature where this enthalpy excess begins to be recovered using a slow heating rate. The enthalpy excesses and the recovery temperatures are found in several glasses [15,16,17,18,19,20,21,22]. The glass phase is stabilized after relaxation below the percolation threshold of broken bonds named configurons, occurring during the first heating at T_g_. The specific heat undergoes a second-order phase transition at T_g_ with critical exponents during heating which are absent during the first cooling [23,24,25,26]. The formation of the glass phase after relaxation and broken bonds during heating is governed by the presence of a new phase, initially called Phase 3, discovered in supercooled water [27,28], having an enthalpy coefficient Δε_lg_ equal to the difference between those of Liquids 1 and 2 and defining the configuron enthalpy (Δε_lg_ H_m_) [29].

The second class of homogenous nucleation reduced temperatures θ_n+_ above θ = 0 respects the relation with the configuron enthalpy coefficient (Δε_lg_ H_m_= θ_n+_ H_m_) and (H_m_) the melting heat of surviving entities and crystals. The configuron phase (Phase 3) undergoes a first-order transition characterized by an endothermic or exothermic latent heat equal to |θ_n +_ H_m_|. Consequently, the relation (−Δε_lg_ = θ_n+_) is also respected because Δε_lg_ can be positive or negative [11]. The reduced temperature θ_n+_ above θ = 0 is equal to the Scher and Zallen percolation threshold of configurons [15,30].

Exothermic transitions are induced below T_m_ during heating or cooling, depending on the heating and cooling rates. Undercooling with or without crystallization is observed during the first cooling depending on the cooling rate. The enthalpy coefficient Δε_lg_, equal to singular values of Phase 3, results from this first-order transition [31]. Heating from the glass state can produce a first-order transition between T_m_ and T_g_ with or without crystallization. The fraction Δε_lg_ is involved in the formation of a liquid glass up to T_n+_ and the crystallized fraction (1 − Δε_lg_) melts at T_m_ [32]. The glass fraction stabilizes nanocrystallization of any material because T_n+_ is higher than T_m_. For example, volcanic rocks are composed of crystals of various sizes surrounded by glasses containing variable fractions of SiO_2_.

A liquid glass is created by a first-order transition of the configuron phase to stabilize its enthalpy to a negative singular value (−Δε_lg_) up to T_n+_ with a specific heat equal to zero. The upper limit of Δε_lg_ is defined by T′_g_/T_m_, the glass transition temperature of the liquid glass being equal to (T′_g_ = 2 T_m_ − T_g_) [33,34].

Our project in this paper is to induce a first-order transition under extreme pressures in NiTi_2_ by molecular dynamics simulations and observe the stabilization of the liquid glass, varying the temperature under constant pressure to induce a memory effect [35]. The choice of NiTi_2_ is guided by molecular dynamics simulations showing that the glass transition occurs at (T_g_ = 800 K) at high cooling rate, revealing structural changes via radial distribution functions at (T_g_) [36].

## 2. Predictions of NCHN and Configuron Models

### 2.1. The Nonclassical Homogeneous Nucleation (NCHN) and the Classical Homogeneous Nucleation (CHN)

In the NCHN equation, the Gibbs free energy change for a nucleus formation in a melt is given by Equation (1) [7]:

(1) $${∆G}_{ls}=\left(\theta -\varepsilon_{ls}\right)H_{m}/V_{m}\times 4\pi R^{3}/3+4\pi R^{2}{(1+\varepsilon_{ls})\sigma }_{ls},$$

where R is the nucleus radius and following Turnbull [1], σ_ls_ is its surface energy for ε_ls_ = 0, given by Equation (2), θ is the reduced temperature (T − T_m_)/T_m_, H_m_ is the enthalpy of melting at T_m,_ and V_m_ is the molar volume:

(2) $$\sigma_{ls}{\left(V_{m}/N_{A}\right)}^{-1/3}=\alpha_{ls}H_{m}/V_{m},$$

The complementary enthalpy ε_ls_ × H_m_/V_m_, introduced in the classical homogeneous nucleation (CHN) equation, authorizes the presence of growth nuclei above T_m_. The prediction of an exothermic or endothermic enthalpy change, at the same temperature T_n+_ above T_m_, viewed as due to the presence of antibonds or bonds, sets the NCHN equation [33]. The CHN equation is obtained for ε_ls_ = 0, and leads to a homogeneous liquid above T_m_ in contradiction with the presence, in the liquid above T_m_, of first-order transitions, and single crystal formations by cooling overheated liquid droplets.

The nucleation rate (Jvt) in a melt of volume v, after a time t, and the thermally activated energy barrier ΔG*_ls_/k_B_T are given in Equations (3) and (4) [4]:

(3) $$(Jvt)=\left(K_{v}vt\right)exp(\frac{-{∆G}_{ls}^{*}}{k_{B}T}),$$

(4) $$\frac{{∆G}_{ls}^{*}}{k_{B}T}=\frac{16\pi S_{m}\alpha_{ls}^{3}}{3N_{A}k_{B}{\left(1+\theta )(\theta -\varepsilon_{ls}\right)}^{2}},$$

where S_m_ is the entropy of melting of crystals and condensed entities [4,7]. The critical parameter ($\frac{{∆G}_{ls}^{*}}{k_{B}T}$) is not infinite at the melting temperature T_m_ when (ε_ls_) is present. In this case, this event now occurs above T_m_ for θ = ε_ls._ The nucleation rate is equal to 1, and Ln(Jvt) = 0 when Equation (5) is respected:

(5) $${∆G}_{ls}^{*}/k_{B}T=ln(K_{v}vt).$$

**Figure 1:**
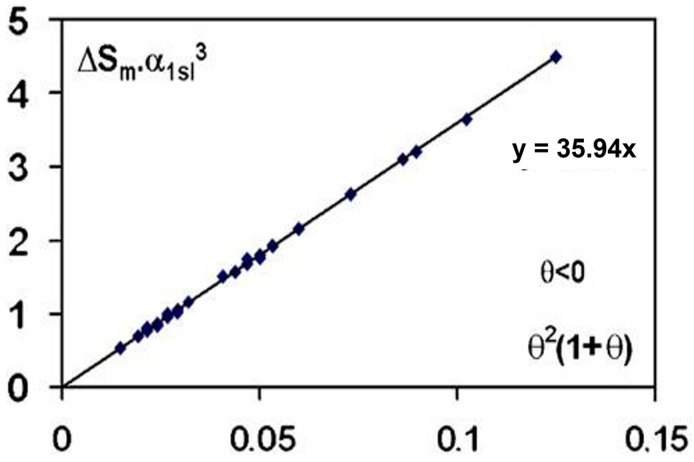
The quantity α^3^S_m_ is plotted as a function of θ^2^(1 + θ), θ being the algebraic lowest undercooling reduced temperature of 38 elements; α_1ls_ and S_m_ are, respectively, the dimensionless surface energy and the entropy of melting in JK^−1^ mole^−1^. The parameters (θ) have been assembled by [4]. Reproduced from [7], Copyright 2007 Elsevier.

The surface energy coefficient α_ls_ in Equation (2) is determined from Equations (3)–(5) and equal to Equation (6):

(6) $$\alpha_{ls}^{3}=\frac{3N_{A}k_{B}\left(1+\theta \right){\left(\theta -\varepsilon_{ls}\right)}^{2}}{16\pi S_{m}}ln(K_{v}vt).$$

(S_m_α_ls_^3^) is effectively proportional to (1 + θ) θ^2^ with (θ) being the reduced temperature of undercooling of 38 liquid elements, obtained after overheating the melt, using volumes v and time t with vt = 10^−8 ± 1^ m^3^s, K_v_ = 90, Ln (K_v_vt) = 71.9 ± 1 in agreement with the predictions of Equation (6), as shown in Figure 1 [4].

This law is beautifully respected in the two nucleation models and is due to homogeneous nucleation phenomena. The phase diagram of liquid elements predicts these homogenous nucleation temperatures [34]. A first-order transition occurs at the undercooling reduced temperature θ. The exothermic enthalpy is either totally attributed to crystallization with CHN without considering any homogeneous nucleation because the liquid is viewed as being homogenous above T_m_, or only partially to the nucleation of a glassy fraction depending on the nucleation temperature of first-order transition with NCHN [29,32,37]. Recent examples of these two approaches are devoted to the undercooling of Pt_57_Cu_23_P_20_ alloy [31,38].

The nucleation temperatures θ_n−_ and θ_n+_ in the NCHN model are obtained for d$\alpha_{ls}^{3}$/dθ = 0 and obey Equation (7):

(7) $$d\alpha_{ls}^{3}/d\theta \sim (\theta_{n+}-\varepsilon_{ls})\left(3\theta_{n-}+2-\varepsilon_{ls}\right)=0.$$

There are two types of homogeneous nucleation temperatures as shown by Equation (7): θ_n−_ = (ε_ls_ − 2)/3 below T_m_, and θ_n+_ = ε_ls_ above T_m_. The first one leads to the glass formation temperature, while the second is a nucleation reduced temperature θ_n+_ above T_m_ obtained in many experiments, observing the undercooling versus the overheating rates of liquid elements, and glass-forming melts [39,40,41,42,43]. These nucleation temperatures predict, for consequence, the existence of second melting temperatures T_n+_ of growth nuclei above T_m_ and their growth below T_m_, shown in several publications [11,32,44,45,46,47,48,49,50,51,52,53,54,55,56,57].

### 2.2. The Enthalpy Coefficients of Two Liquid States Depending on the Glass Transition Temperature at T_g_ below T_m_

The NCHN model describes two liquid states characterized by enthalpy coefficients. The coefficient ε_ls_ of the initial liquid state called Liquid 1 is a quadratic function of Equation θ in Equation (8) [7]:

(8) $$\varepsilon_{ls}=\varepsilon_{ls0}(1-\theta^{2}/\theta_{0m}^{2}),$$

where θ_0m_ is the Vogel–Fulcher–Tamann (VFT) reduced temperature leading to ε_ls_ = 0 for θ = θ_0m_ [8]. (θ^2^_0m_), minimizing the complementary surface energy, is given in Equation (9) [9]:

(9) $$\theta_{0m}^{2}=\frac{8}{9}\varepsilon_{ls0}-\frac{4}{9}\varepsilon_{ls0}^{2},$$

with $\varepsilon_{ls0}=\theta_{g}+2$.

The glass transition gives rise to Liquid 2 with a new coefficient ε_gs_, also depending on θ^2^ in Equation (10) with the second VFT reduced temperature (θ_0g_) given in Equation (11):

(10) $$\varepsilon_{gs}=\varepsilon_{gs0}(1-\theta^{2}/\theta_{0g}^{2}),$$

(11) $$\theta_{0g}^{2}=\frac{8}{9}\varepsilon_{gs0}-\frac{4}{9}\varepsilon_{gs0}^{2},$$

with $\varepsilon_{gs0}=1.5\theta_{g}+2$, as expected with Equation (7), θ_g_ is the reduced glass transition temperature, and (ε_ls0_) and (ε_gs0_) are values for which the activation energies of Liquids 1 and 2 are equal at T_g_, leading to an activation energy equal to zero for the Liquid 3 characterized by Δε_lg_ = (ε_ls_ − ε_gs_) [29].

### 2.3. Formation of New Phases through First-Order Transitions below T_m_

First-order transitions are observed below T_m_, depending on the heating rate. They lead to new glassy phases having a weaker enthalpy than that of the initial glass state, which is equal to zero. The enthalpy coefficient Δε_lg_ is equal to −Δε after the transition, and the first-order transition reduced temperature θ_x_ is determined with Equation (12):

(12) $$\theta_{x}=\left(-3\pm {\left[9-\frac{4\left(2-\varepsilon_{gs0}-∆\varepsilon \right)\varepsilon_{gs0}}{\theta_{0g}^{2}}\right]}^{0.5}\right)\frac{\theta_{0g}^{2}}{2\varepsilon_{gs0}}$$

where ε_gs0_ is the enthalpy coefficient of Liquid 2 at θ = 0 given in Equation (10). This first-order reduced temperature is called θ_x_, even if it is not accompanied by crystallization.

This equation is used after application of very high temperatures, and the undercooling reduced temperature is directly equal to θ_x_ and Δε_lg_ = −Δε (see for example [31]).

### 2.4. The Glassy State of Phase 3 Up to the Melting Temperature T_n+_ above T_m_

The difference Δε_lg_ in Equation (13) between the coefficients ε_ls_ and ε_gs_ determines the enthalpy coefficient of Phase 3 during heating and the configuron enthalpies when the quenched liquid has escaped crystallization:

(13) ∆εlgθ=εls−εgs=[εls0−εgs0−∆ε−θ2εls0θ0m2−εgs0θ0g2],

This phase explains the homogenous nucleation of phase transformations in supercooled water [28], and corresponds to the enthalpy coefficients of broken bonds (configurons) at the glass transition [29].

In the absence of first-order transition below T_m_ during the first heating, Phase 3 undergoes an exothermic transition at a reduced temperature θ_n+_ = Δε_lg_, respecting Equation (7) above T_m_. This transition occurs at T_n+_ ~ 1.145 T_m_ and corresponds to the temperature where the liquid viscosity ceases to respect the Arrhenius law during cooling from high temperature [29,58]. Rapidly quenched glass formers are amorphous and are transformed into glass phases by relaxing enthalpy during the first heating. Two liquids give rise, at first, to an intermediate Phase 3 below T_3_ < T_g_ respecting the entropy constraints, and then, the enthalpy increases towards that of the glass phase up to T_g_. Phase 3 carries a medium-range order above T_g_, which can be superheated above the melting temperature up to T_n+_. The configuron model is successfully applied to 54 glasses, explaining the transitions at T_n+_ by percolation and the existence of a glassy ‘ordered’ fraction equal to the critical threshold of Scher and Zallen (Φ_c_ = 0.15 ± 0.01), producing T_g_ [25,30]. The temperature T_n+_ is the melting temperature of residual bonds involved in this glassy fraction [29].

First-order transitions occur in undercooled liquids at θ_x_ below T_m_, depending on the heating rate, producing an exothermic heat equal to −Δε H_m_ for ε_gs_ = 0 in Equation (12), without crystallization [11,34,50,59,60,61,62]. The coefficient Δε is equal to singular values of the vitreous state enthalpy coefficient: Δε_lg_ (θ_0m_), ε_gs0_, ε_gs0_/2, θ_n+_ ≅ 0.145, and 0. This enthalpy is recovered at various reduced temperatures θ_n+_ = Δε, including Δε ≅ 0.145. Two Phase 3 states with opposite enthalpies disappear at the same reduced temperature θ_n+_ = 0.145 and could contain clusters-bound colloids with two opposite values of enthalpy corresponding to antibond and bond breaking. All colloid bonds disconnect at T_n+_ > T_m_ and give rise in congruent materials, through a first-order transition at T_n+_, forming a liquid, containing tiny superatoms, built by short-range order [33]. This description is compatible with the presence of microheterogeneities in metallic overheated liquids, as already observed by Popel et al. [45,63].

The first-order transition at θ_x_ induces either an enthalpy of Phase 3 being constant and equal to −Δε H_m_ between θ_x_ and θ_n+_ at high rates of cooling without crystallization, or with a crystallized (or condensed) fraction (1 − Δε) melted at T_m_. For a fully crystallized volume, the enthalpy of crystals is assumed to be −H_m_, considering that the denser crystalline phases lead to Δε = 1 [64]. In the second case, the melt above T_m_ contains a glassy fraction Δε and a homogeneous liquid fraction equal to (1 − Δε). This mixed state leads to the formation of crystals, coexisting with a glass fraction up to T_m_ with a glass state stable up to T_n+_ [11,26,31,32,33].

### 2.5. The Formation of a Liquid Glass above T_m_

The formation of a liquid glass is expected at a temperature (T′_g_ = 2 T_m_ − T_g_) [32]. A glass transition at θ_g_ is accompanied at high temperatures by a second glass transition occurring at −θ_g_ above T_m_. This phenomenon is a consequence of the θ^2^ dependence of the enthalpy of glass phases. The corresponding VFT temperature squares are negative at high temperatures and the values of ε_gs0_ and ε_ls0_ become higher than 2. These glass transitions have been already observed three times without being recognized as liquid glasses by the experimentalists [65,66,67,68]. The two transitions occur at T_g_ = 715 K and T′_g_ = 1535 K in Cu_46_Zr_46_Al_8_ with T_m_ = 1125 K, during cooling of the melt, accompanied by an exothermic transition [32,65]. In the second material, the composite (ZIF-62) (Al-rich) (50/50) has a solidus melting temperature at 650 K, T_g_ = 591 K and T′_g_ = 709 K observed during heating and accompanied by an endothermic latent heat. A glass transition still exists in tin far above T_m_, and looks like a partial glassy fraction by [34,68]. A liquid glass is observed in suspensions of ellipsoidal colloids [69].

### 2.6. Validation of NCHN with Molecular Dynamics Simulations

A phase diagram of ice in single-walled carbon nanotubes at atmospheric pressure is established by numerical simulations, and predicts ice melting points as a function of their diameter up to 1.7 nm [70]. All of these melting points agree with the temperatures T_n+_ given by the NCHN equation [37]. A first-order transition from liquid to homogenous glass, denoted L-glass, is predicted with the NCHN equation since 2016, in liquid elements, having a Lindemann constant close to 0.103, accompanied by a latent heat of 10.5% of the melting heat [10]. The transition under pressure of ^4^He is the first example of this phenomenon [71]. Recent molecular dynamics simulations identify, in addition, a first-order transition at T_x_ from liquid (L) to a metastable heterogenous solid-like phase, denoted as G-glass, when a supercooled liquid evolves isothermally below its melting temperature T_m_ at deep undercooling [59,61]. The NCHN model describes the first-order transitions from liquid to L-glass and to G-glass in agreement with these simulations [62,72]. The G-glass is a heterogenous phase consisting of regions fully embedded in a surrounding disordered medium.

Molecular dynamics simulations show full melting at T_n+_ = 1.119 T_m_ for Zr [60], 1.126 T_m_ for Ag [61], 1.219 T_m_ for Fe, and 1.354 T_m_ for Cu [73]. The NCHN model applied to liquid elements is based on the increase of the Lindemann coefficient with the heating rate. The glass transition at T_g_ and the nucleation temperatures of G-phases at T_x_ and their melting at T_n+_ are predicted. A universal law relating T_n+_ and T_x_ shows that T_x_ cannot be higher than 1.293 T_m_ for T_n+_ = 1.47 T_m_. The enthalpies of G-phases have singular values, corresponding to the increase of percolation thresholds with T_g_ and T_x_ above the Scher and Zallen invariant at various heating and cooling rates [62].

As a conclusion of this chapter, the liquid glass states extend far above T_m_. Varying pressures and temperatures and formation conditions of liquid glasses could be determined. For that purpose, a study of glass states and liquid glass states of (NiTi_2_) [36,74] under pressure by molecular dynamics simulations is developed in the next chapter.

## 3. Numerical Simulations

Molecular dynamics (MD) computer simulation using a open source code – software package for classical molecular dynamics Large-scale Atomic/Molecular Massively Parallel Simulator (LAMMPS, https://www.lammps.org/#gsc.tab=0 accessed on 29 October 2020) at periodic boundary conditions was used for modeling [75]. The simulation was performed at a 1 fs time step using the embedded atom potential derived for Ti–Ni alloys [76]. An atomic cell containing 128,000 atoms was heated to 2500 K to melt, and then cooled down to produce a glass. Melting was confirmed by the radial distribution function and stabilization of the density variation with time. A thermostat was used to control the temperature [77,78], while pressure was maintained by a barostat [79].

Deviations and irreproducibility have been studied using these types of coupling to determine possible deviations in dynamic properties for time constants as low as 0.01 ps. Reliable and thermostable dynamic properties can be derived for coupling time constants above 0.1 ps [80]. The atomic volume is calculated here using coupling time constants much higher than 10^−13^ s. The weakest time (10^−13^ s) would correspond to a temperature variation of 1 K without a change of dV_at_/dT. The highest time used in these simulations is 3 × 10^−10^ s. The atomic volume variation under a pressure of 1000 GPa is dV_at_/dp = 1.7881 × 10^−7^ with p in bars at a constant temperature of 299.7 K. The typical pressure variation is ~10,000 bars, leading to ΔV_at_ = 1.788 × 10^−3^ Å^3^. Changing the temperature of 1 K has no influence on dV_at_/dp. Typical variations (ΔT) at ambient pressure are ~5 K. The reproducibility of the simulation during several cycles of temperature and pressure shows that the limit of ~10^−13^ s has no influence on the results because the times of coupling are much higher than 0.1 ps.

## 4. NiTi_2_, a New Liquid Glass

### 4.1. The Vitreous States of NiTi_2_ with T_g_ = 800 K and 695.5 K

The glass transition of NiTi_2_ is equal to 800 K with a cooling rate of 10^12^−10^13^ K/s and ~700 K [36] for an experimental value obtained on heating [74]. NiTi_2_ is chosen because its glass transition, at T_g_ = 800 K, and at high cooling rate, was initially determined by molecular dynamics simulations [36].

**Figure 2:**
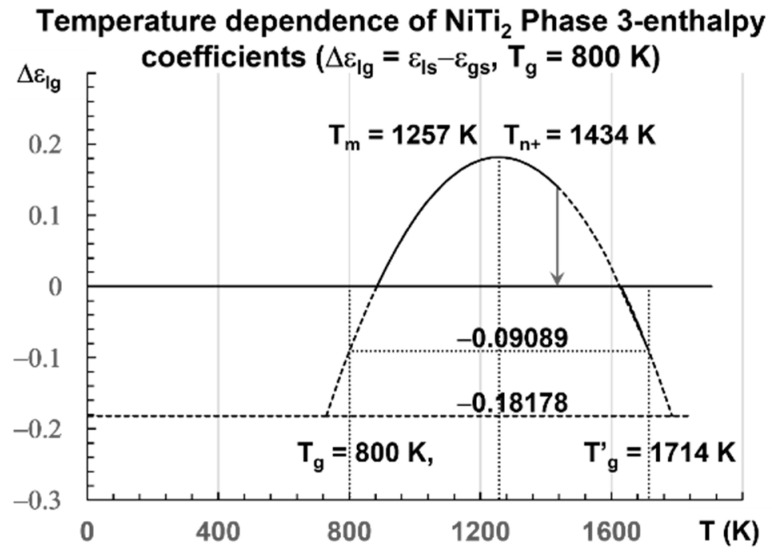
Temperature dependence of NiTi_2_ configuron enthalpy for T_g_ = 800 K. The enthalpy coefficient (Δε_lg_) of Phase 3 given in Equation (13) is plotted as a function of the temperature in Kelvin for a fragile liquid with T_g_ = 800 K. The singular values (Δε_lg_) = −Δε_gs0_ = −0.18178 of Phase 3 and zero in the glass state are observed. The two others Δε_gs0_/2 = 0.09089 and Δε_lg_(θ_0m_) = 0.36369 are not obtained at room temperature. An exothermic first-order transition is expected at T_n+_ = 1434 K with θ_n+_ = Δε_lg_ = 0.14085 at a low heating rate.

The enthalpy coefficients calculated with Equations (7)–(13), representing the enthalpy coefficient of Phase 3 in Figure 2, are given below in Equation (14), and are those of a fragile glass with T_m_ = 1257 K [80] and T_g_ = 800 K:

(14) $$\theta_{g}=(800-1257)/1257=-0.36356;\;\varepsilon_{\mathrm{lso}}=\theta_{g}+2=1.63644;\;\varepsilon {\mathrm{gs}}_{0}=1.5\theta_{g}+2=\;1.45466,\;\theta_{0m}^{2}=\frac{8}{9}\varepsilon_{lso}-\frac{4}{9}\varepsilon_{lso}^{2}=0.264422;\;\theta_{0g}^{2}=\frac{8}{9}\varepsilon_{gs0}-\frac{4}{9}\varepsilon_{gs0}^{2}=0.352573;\;\;\mathrm{Liquid}\;1:\;\varepsilon_{ls}=1.63644(1-\theta^{2}/0.264422);\;\mathrm{Liquid}\;2:\;\varepsilon_{gs}=1.45465(1-\;\theta^{2}/0.352573);\;\mathrm{Phase}\;3:\;{∆\varepsilon }_{lg}=\varepsilon_{ls}-\varepsilon_{gs}=0.18178-2.0629\times \theta^{2}$$

The singular values of ${∆\varepsilon }_{lg}$ are calculated with Equation (14) for various values of θ: Δε_gso_ = 0.18178 (θ = 0), Δε_gso_/2 = 0.09089 (θ = θ_g_), ${∆\varepsilon }_{lg}\left(\theta_{0m}\right)=0.36369\;\theta_{0m}^{2}$ = 0.264422), Δε_lg_ = 0.14085 for θ = 0.14085, and Δε_lg_ = 0 (θ = −0.29685). They are the enthalpy coefficients of the configuron phase which are determined during the first heating after the first cooling from a temperature higher than T_m_.

The liquid glass transition would be $T_{g}^{\prime }=2T_{m}-T_{g}=1714K(\theta_{g}^{\prime }=+0.36356)$ at the end of the first heating after the formation of broken bonds (configurons).

The enthalpy coefficient (Δε_lg_) of Phase 3 is −0.18178 for T_g_ = 800 K [36] with cooling rates of 10^12^−10^13^ K/s and −0.22335 for T_g_ = 695.5 K at a low heating rate [74]. A transition is expected at T_n+_ = 1434 K for a liquid with T_g_ = 800 K, being quenched from temperatures near T_m_ and heated at 0.3 K/s [32]. Many exothermic or endothermic transitions have already been observed at these temperatures [42,44,45,46,48,52,53,54]. Recent study confirms the existence of these temperatures T_n+_ > T_m_ in all liquids previously vitrified at low temperatures [29]. All these transitions correspond to the temperature below which the Arrhenius law is no longer working, occurring at T_n+_ ≅ 1.145 T_m_ [58]. They are glass transitions of the fraction (≅0.1445) of unbroken bonds, equal to the percolation threshold predicted by the configuron model and the Scherr and Zallen invariant [25,26].

**Figure 3:**
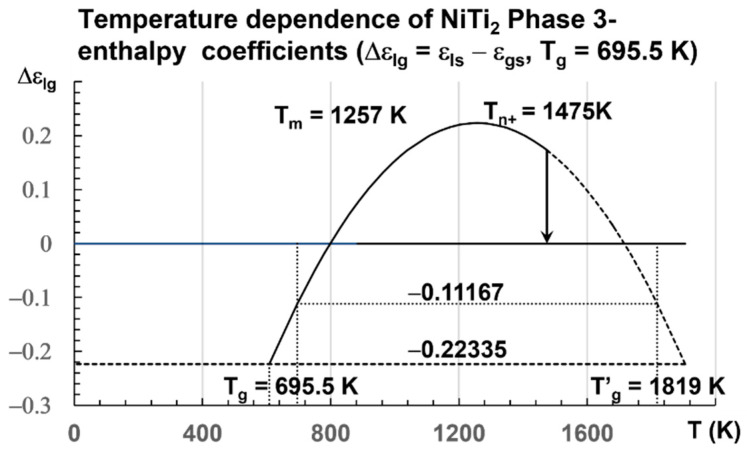
Temperature dependence of NiTi_2_ configuron enthalpy for T_g_ = 695.5 K. The enthalpy coefficient (Δε_lg_) of Phase 3 given in Equation (15) is plotted as a function of the temperature in Kelvin for a fragile liquid with T_g_ = 695.5 K. The singular values (Δε_lg_) = −Δε_gs0_ = −0.22335 of Phase 3 and zero of the glass state are observed. The two others Δε_gs0_/2 = 0.11167 and Δε_lg_(θ_0m_) = 0.29442 are not obtained at room temperature. An exothermic first-order transition is expected at T_n+_ = 1475 K with θ_n+_ = Δε_lg_ = 0.17306 at a low heating rate.

The enthalpy coefficients for T_g_ = 695.5 K and T_m_ = 1257 K, used in Figure 3, are given below in Equation (15) [8,32,74]:

(15) $$\theta_{g}\;(695.5-1257)/1257=-0.446698;\;\varepsilon_{\mathrm{lso}}=\theta_{g}+2=1.55330;\;\varepsilon {\mathrm{gs}}_{0}=1.5\;\theta_{g}+2=\;1.32995,\;\theta_{0m}^{2}=\frac{8}{9}\varepsilon_{lso}-\frac{4}{9}\varepsilon_{lso}^{2}=0.388381;\;\theta_{0g}^{2}=\frac{8}{9}\varepsilon_{gs0}-\frac{4}{9}\varepsilon_{gs0}^{2}$$

=0.396058 [8], Liquid 1: εls = 1.55330(1 − θ^2^/0.388381), Liquid 2: ε_gs_ = 1.553302(1 − θ^2^/0.396058), Phase 3: ∆ε_lg_(θ) = ε_ls_ − ε_gs_ = 0.22335 − 1.679 × θ^2^; T_g_ = 695.5 K [74] and θ_g_ = −0.446698 and T′_g_ = 2 T_m_ − T_g_ = 1818.5 K and θ′_g_ = 0.446698 [32].

A transition is expected at slow heating at T_n+_ = 1475 K for a liquid being quenched from temperatures much closer to T_m_. The enthalpy coefficient of Phase 3 is −0.22335. The volume change calculated by numerical simulations is equal to 0.22335 × 1.5661 = 0.3498 ± 2%. The coefficient 1.5661 ± 1% transforms the enthalpy coefficient −0.22335 in a volume change in Å^3^ at the melting temperature T_m_ because the enthalpy of melting is equal to the enthalpy variation between T_m_ and 2 T_m_ [34]. Simulations of the volume are used to predict this coefficient of 1.5661 ± 1%. The transition at T_n+_ is expected at 1475 K, a temperature where θ_n+_ = Δε_lg_ = 0.17306.

### 4.2. The Atomic Volume Hysteresis above the Melting Temperature Up to 2120 K

**Figure 4:**
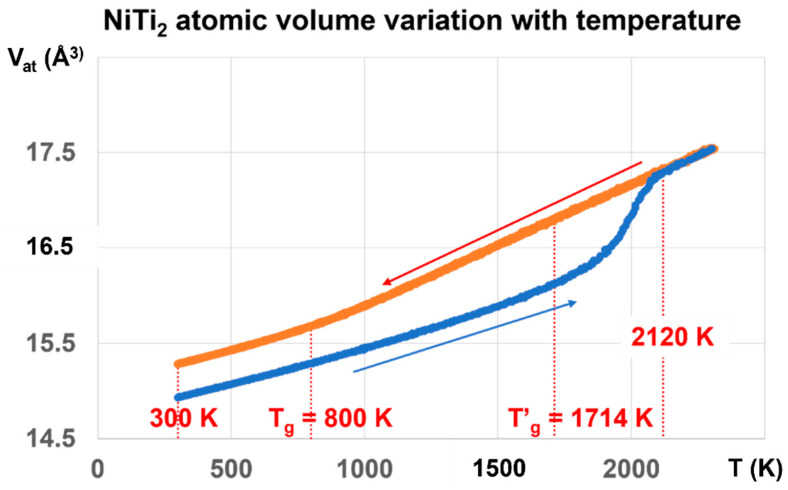
NiTi_2_ atomic volume variation with temperature using cooling and heating rates of 10^13^ K/s. Two values of the atomic volume are observed and fixed by the thermal history under pressure. The red line is obtained by decreasing the temperature from 3000 K down to 300 K under pressures weaker than 700 bars. The first glass transition occurs at 800 K during cooling. The melt is submitted to a glass transition at 2120 K during heating. The atomic volume along the blue line is very stable from 300 to 800 K, whatever the pressure of ±700 bars is, and results from a first-order transition, under a pressure of 1000 GPa at 1000 K, varying the temperature to 300 K, and finally decreasing the pressure to 1 bar. The glass phase totally disappears at 2120 K instead of 1714 K.

The NiTi_2_ atomic volume V_at_ in Angström^3^, represented by the red line in Figure 4, is obtained by cooling the melt from 3000 K to 300 K, and is equal to 15.2829 Å^3^ at 300 K.

The atomic volume represented by the blue line starts at 300 K from V_at_ = 14.9331 Å^3^ obtained after applying a pressure of 1000 GPa at 1000 K, lowering the temperature down to 300 K, and finally decreasing the pressure to 1 bar. The hysteresis cycle between 300 and 3000 K is measured under weak pressures P, varying between −660 bar and +700 bar. The line thicknesses include the volume variations with pressure. The atomic volume follows the blue line during reheating up to 2120 K. Above this temperature, the blue and red lines merge, and the volume is reproducible with pressure increase or decrease. The glass transition temperature occurs at 2120 K without volume jump.

The temperature T′_g_ = 1714 K is equal to (2 T_m_ − T_g_) with T_m_ = 1257 K and T_g_ = 800 K. The temperature of 2120 K corresponds to θ_n+_ = 0.68632, being equal to the sum of singular enthalpy coefficients (0.14085 + 0.18178 + 0.36369) of Phase 3 with T_g_ = 800 K, and determining the highest glass transition temperature of Phase 3 and the highest melting temperature of NiTi_2_, as already observed for several liquid elements [62].

The first-order transition of the glass phase expected at T′_g_ = 1714 K is not observed by heating from low temperatures. Nevertheless, ΔV_at_, the difference between the red and blue lines, is maximum at this temperature and decreases to zero from 1714 K to 2120 K. In addition, it is constant from 300 K up to the first glass transition at T_g_ = 800 K where ΔV_at_ begins to increase with the temperature.

The glass transition at 800 K is observed on the red curve by the slope change of V_at_ at this temperature while a new glass state is induced by a first-order transition, occurring at 1000 GPa by decreasing the temperature from 1000 to 300 K, followed by lowering the pressure down to 1 bar. This new glass state disappears during heating at 2120 K. The NiTi_2_ melt cooled from 3000 K to 800 K does not undergo liquid glass transformation.

### 4.3. The First-Order Transition under 1000 GPa

**Figure 5:**
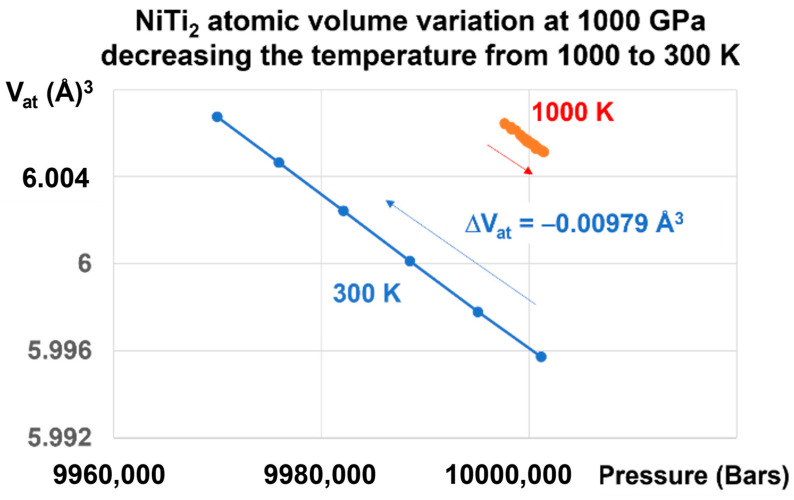
NiTi_2_ atomic volume variation at 1000 GPa, decreasing the temperature from 1000 to 300 K. Increasing the pressure at T = 1000 K up to 1000 GPa and lowering the temperature down to 300 K induces a first-order transition in the glass state accompanied by a volume change equal to ΔV = −0.00979 Å^3^.

A liquid glass state is revealed in Figure 5 by a first-order transition as expected. An atomic volume change, equal to −0.00979 Å^3^ under 1000 GPa, is induced by a reduction of temperature from 1000 K to 300 K and by the memory of this transition, as shown in Figure 4.

A memory effect has been numerically predicted in a three-dimensional model for structural glass when submitted to a temperature cycle [35]. Here, a first-order transition is observed and builds a memory effect.

**Figure 6:**
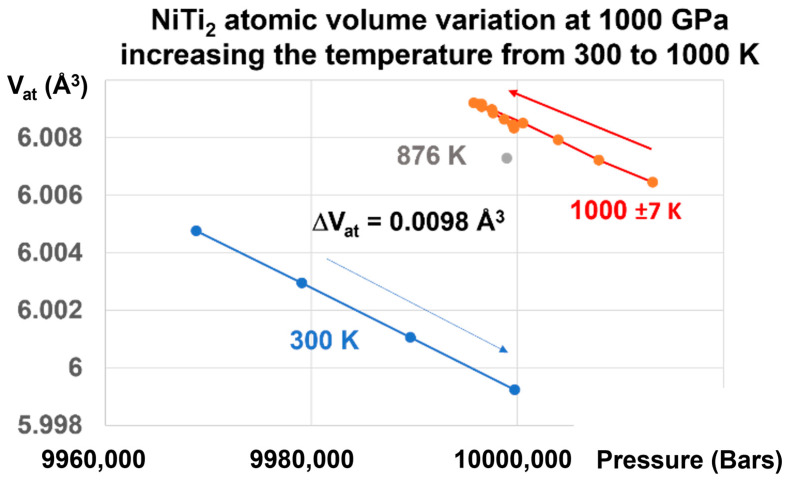
NiTi_2_ atomic volume variation at 1000 GPa, increasing the temperature from 300 to 1000 K. Increasing the pressure on the glass state at T = 300 K up to 1000 GPa and increasing the temperature from 300 K to 1000 K induces a first-order transition from the glass to the liquid state, and a volume change of +0.00980 Å^3^, equal to that observed in Figure 5.

This transition is reversible, as shown in Figure 6.

Applying a pressure of 1000 GPa at 300 K and increasing the temperature to 1000 K induces an increase of the atomic volume of 0.0098 Å^3^, as shown in Figure 6. This transformation reduces the difference (ΔV_at_) = −0.3498 Å^3^ at 300 K, as shown in Figure 4, to a value which is 35.69 times weaker. This factor (35.69) is related to the increase of the melting heat under pressure.

### 4.4. The Phase Diagram of Glass Phases in Fragile Liquids

**Figure 7:**
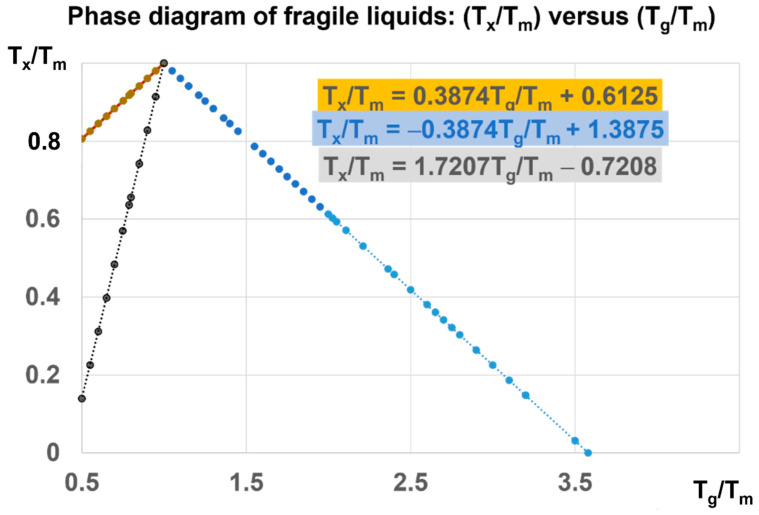
Phase diagram of fragile liquids: (T_x_/T_m_) versus (T_g_/T_m_). For each value of T_x_/T_m_, higher than 0.8 at zero pressure, there are two glass transitions corresponding to T_g_ and T′_g_. For T_x_/T_m_ > 0.13962, there is a unique glass transition T′_g_/T_m_ < 3.22076. A pressure has, for consequence, to reduce T_x_/T_m_ and to increase T_g_/T_m_ beyond 3.22076. This ratio tends to the upper limit equal to 3.581 when the pressure tends to infinite.

The phase diagram in Figure 7 is devoted to Phase 3 of fragile liquids, having a melting temperature (T_m_) and submitted to first-order transitions induced at T_x_/T_m_ and T_g_/T_m_ at various heating rates from their low temperature glass state. The ratio of T_g_/T_m_ of fragile glasses is always higher than 0.5 (θ_g_ > −0.5). This limit corresponds to Vogel–Fulcher–Tamann temperatures higher than T_m_/3. The reduced glass transition at θ_g_ is varying from −0.5 to 2.581 and T_g_/T_m_ from 0.5 to 3.581. For each value of θ_g_, there are first-order transitions of Phase 3, occurring at θ_x_ and θ_n+_, accompanied by enthalpy coefficient changes respectively equal to −Δε and +Δε. For example, T_x_/T_m_ = 0.22335 = Δε leads to two glass transitions at T′_g_/T_m_ = 0.5487 and 3 (θ_g_ = −0.77665 and 2) at zero pressure and constant melting temperature.

There are two types of first-order transitions at T_x_. In the first case, the Phase 3 enthalpy falls to −H_m_ at T_x_, remains constant up to T_m_, and an enthalpy fraction Δε is recovered through a new first-order transition at T_n+_ far above T_m_. Consequently, the melting enthalpy, recovered at T_m_, is reduced and equal to (1 − Δε) H_m_. The temperature (T_n+_), corresponding to a reduced temperature equal to θ_n+_ = Δε, is the glass transition reduced temperature of the liquid fraction Δε, which is not crystallized or condensed at T_x_ [31,34]. This diagram is also used to predict the glass transitions under pressure, taking account of the pressure dependence of T_m_ and H_m_.

In the second case, the transition at T_x_ is not accompanied by a crystallized or a condensed fraction, and is only due to the formation of various Phases 3, having enthalpies equal to −Δε H_m_ with Δε also equal to singular values of percolation thresholds of configurons in Equation (14): Δε_sg0_ (θ = 0), Δε_sg0_/2 (θ = θ_g_), Δε_lg_ (θ = θ_0m_), Δε_lg_ (θ_n+_), and zero. There is no crystalline phase. The volume difference ΔV_at_ increases with temperature up to T′_g_ = 1714 K. The liquid fraction [Δε = (T′_g_ − T_m_)/T_m_] participates to the glass state up to T′_g_.

The enthalpy changes, associated with the first-order transitions at T_n+_ = T_g_ and T_x_, are, respectively, equal to ±Δε H_m_. The melting heat H_m_ increases with pressure, while (Δε) decreases as 1/H_m_. The value of Δε is divided by 35.69 under a pressure of 1000 GPa because the melting heat is multiplied by 35.69. Consequently, (Δε) = 0.0098 at 1000 GPa leads to T_g_/T_m_ = (3.581 − 0.0098/0.581) = 3.5641 near the upper limit of 3.581, as shown in Figure 7.

### 4.5. The Melting Temperature Increases with Pressure

The melting entropy is 9.3 J/K/mole in many metals, and the melting heat is quasi-proportional to T_m_ [4]. The pressure application increases the liquid entropy proportionally to the induced melting temperature. Consequently, the melting heat is expected to be proportional to T_m_^2^ and a linear function of the pressure (P), as shown for NiTi_2_ in the following equations:

assuming Δε = 0.22335 for T_g_ = 695.5 K

(16) $${\left(\frac{T_{m}}{T_{0}}\right)}^{2}=1+34.69/1000\times P(GPa)$$

and assuming Δε = 0.18178 for T_g_ = 800 K

(17) $${\left(\frac{T_{m}}{T_{0}}\right)}^{2}=1+28.08/1000\times P(GPa)$$

where T_0_ is the melting temperature at ambient pressure and P is the pressure in GPa. The equation for T_g_ = 695.5 K works and results from the decrease of T_g_ from 800 K to 695.5 K at ambient temperature and pressure. The glass transition temperature at the pressure of 1000 GPa is T_g_ = 800 K. Then, the most probable melting temperature is T_m_ = 6779 K at this pressure, given by Equation (17) for T_g_ = 800 K.

At P = 0, T_m_ is equal to T_0_ = 1257 K [80]. For P =1000 GPa, (T_m_/T_0_)^2^ = 35.69 or 29.08, in agreement with the numerical simulations. (T_m_) under P = 1000 GPa is 7509 K or 6779 K. The dependence of T_m_ with P is given in Table 1, using the two values (0.22335) and (0.18178) of Δε. For P = 500 GPa, Δε and ΔV_at_ are multiplied by 1.948 compared to ΔV_at_ at 1000 GPa. The volume change, calculated at 1000 GPa, is 2% or 3.6% weaker than the simulated value (0.00979). The glass transition even increases at pressures much higher than 1000 GPa, with ΔV_at_ tending to zero.

**Figure 8:**
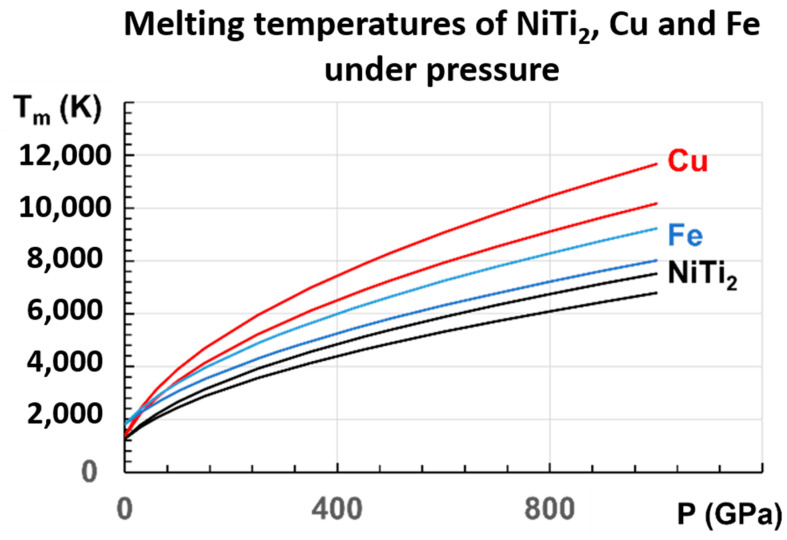
The melting temperatures of Cu, Fe, and NiTi_2_ are represented versus P (GPa), following the proposed equations: for Cu: (18,19); for Fe: (20,21); for NiTi_2_: (16,17).

This linear law of variation with T_m_^2^ with pressure has never been used. It is then important to apply it to other known metals. Copper and iron are chosen. There are many measurements of copper at low pressures [81,82,83,84,85]. The initial slope varies between 36.4 and 47.7 K/GPa. These measurements lead to the two following laws, Equations (18) and (19) for copper, in good agreement with many experimental and theoretical results:

(18) $${\left(\frac{T_{m}}{T_{0}}\right)}^{2}=1+34.69/1000\times P(GPa),$$

(19) $${\left(\frac{T_{m}}{T_{0}}\right)}^{2}=1+47.7/1000\times P(GPa),$$

which are represented in Figure 8, together with those of NiTi_2_ and Fe. For Cu, our extrapolated values in 500 GPa are 7253 K and 8299 K, in agreement with T_m_ = 7900 K [82]. From another determination in 100 GPa, T_m_ = 3900 K [86], and our values are 3463 K and 3905 K.

For Fe, recent measurements up to 103 GPa are determined by X-ray absorption spectroscopy up to 103 GPa, and used to predict a melting temperature of 4850 ± 200 K at the inner core boundary (ICB) of the earth for P = 330 GPa [87]. Another melting temperature of 5500 ± 220 K at the ICB has been extrapolated from a measurement under P = 290 GPa in a resistance-heated diamond-anvil cell [88].

An extrapolation of this experimental result yields a melting point of 5500 ± 220 K at the ICB, higher than the previous reported result. Two laws, Equations (20) and (21) for iron, are deduced from our model, respectively, based on the works of Aquilanti et al. [87] and Sinmyo et al. [88].

(20) $${\left(\frac{T_{m}}{T_{0}}\right)}^{2}=1+18.56/1000\times P(GPa),$$

(21) $${\left(\frac{T_{m}}{T_{0}}\right)}^{2}=1+24.92/1000\times P(GPa),$$

Our extrapolation, at 330 GPa from measurements below 103 GPa, leads to T_m_ = 4834 K, in agreement with the evaluation of 4850 ± 200 K [88].

### 4.6. Increase of the Enthalpy Difference between Liquid and Glass with Temperature from 300 K to 1734 K

**Figure 9:**
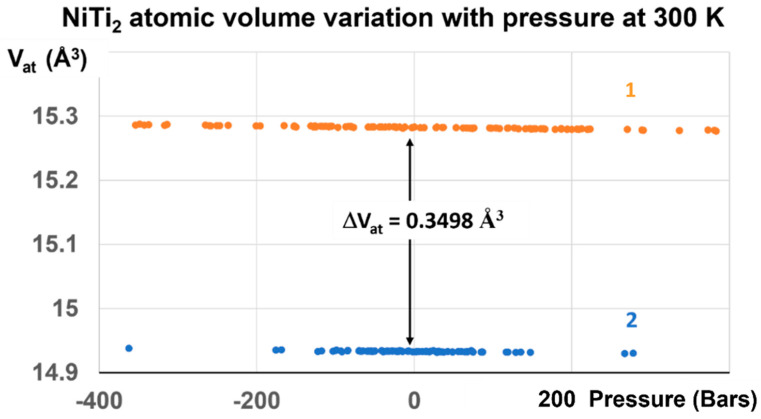
NiTi_2_ atomic volume variation with pressure at 300 K. Two stable volumes at room temperature and under atmospheric pressure are obtained after the first-order transition induced at 1000 GPa. The volume difference ΔV_at_ at 300 K between liquid, noted 1, and glass, noted 2, is equal to 0.3498 ± 0.004 Å^3^, and corresponds to that of configurons for T_g_ = 695.5 K.

The difference ΔV_at_ is equal to 0.3498 Å^3^ under pressure variations between ±700 Bars from 300 K to 700–800 K, as shown in Figure 4 and Figure 9.

**Figure 10:**
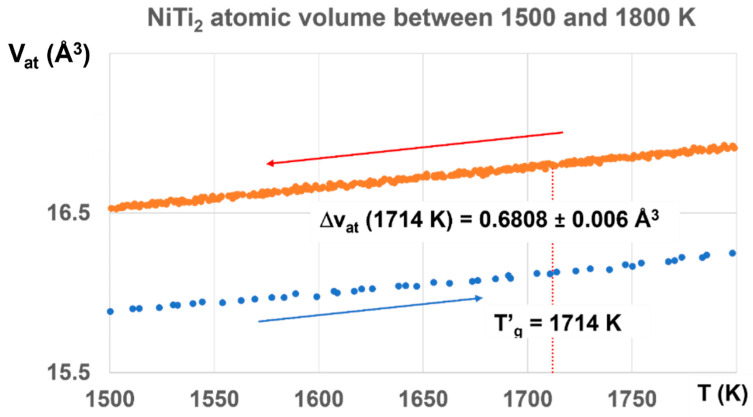
NiTi_2_ atomic volumes between 1500 and 1800 K. The atomic volume difference increases with temperature up to ΔV_at_ = 0.6808, corresponding to Δε = 0.6808/1.5661 = 0.4387, a value a little weaker than T′_g_/T_m_ = 1818.5/1257 = 0.4467, and much higher than T′_g_/T_m_ = 1714/1257 = 0.36356 (q′_g_ = 0.36356). The melt has a glass transition temperature much closer at T′_g_ = 1818.5 K (T_g_ = 695.5 K) than T′_g_ = 1714 K (T_g_ = 800 K).

The glass state, induced by the first-order transition, exists up to T′_g_ =1714 K for T_g_ = 800 K with ΔV_at_, increasing from 0.3498 to 0.6808 Å^3^, as shown in Figure 10.

The value of ΔV_at_ at T′_g_ = 1714 K would correspond to a first-order transition under 1000 GPa induced by a temperature increase from 300 to 1724 K with ΔV_at_ = 0.6808/36.73 = 0.0185 ± 0.0016. This continuous increase of ΔV_at_ up to T′_g_ indicates that a high fraction volume of the sample belongs to a glass phase.

**Figure 11:**
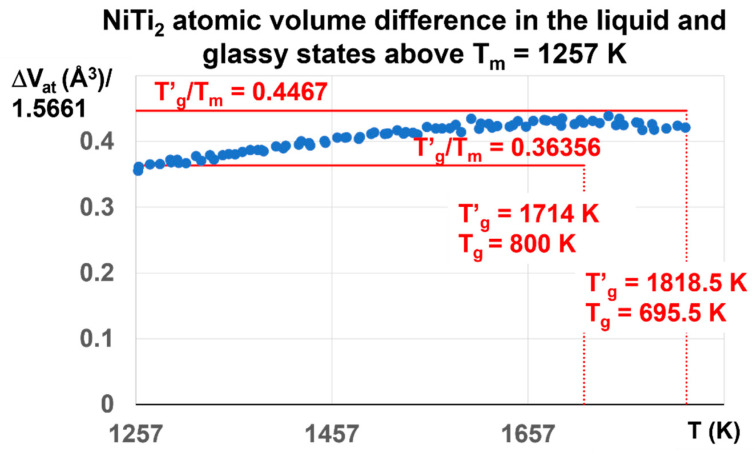
NiTi_2_ atomic volume difference between liquid and glass above T_m_ = 1257 K. The initial liquid glass state at room temperature is characterized by T′_g_ = 1818.5 K and T_g_ = 695.5 K. Its enthalpy coefficient is expected to be equal 0.4467. The maximum calculated value is 0.4387 ± 0.01 at 1714 K. It begins to decrease and attain 0.36356 at T = 1931 K and zero at 2120 K in Figure 3. The influence of the glass phase with T′_g_ = 1818.5 still exists up to 1931 K. The glass phase disappears without first-order transition at 2120 K due to the prolongation of the glass state.

The enthalpy coefficient of Phase 3 with ΔV_at_ = 0.6808 corresponds in fact to Δε = 0.6808/1.5661 = 0.4387, a value a little weaker than θ′_g_ = (1818.5 − 1257)/1257 = 0.4467, and much higher than θ′_g_ = (1714 − 1257)/1257 = 0.36356. The melt has a glass transition temperature much closer at T′_g_ = 1818.5 K (T_g_ = 695.5 K) than T′_g_ = 1714 K (T_g_ = 800 K). The coefficient Δε tends to decrease toward 0.36356, corresponding to T′_g_ = 1714 K and T_g_ = 800 K, as shown in Figure 11.

The atomic volume change at the melting temperature T_m_ = 1257 K is equal to the volume variation (ΔV = 1.5661 Å^3^) of the melt between T_m_ and 2 T_m_ [34]. A new liquid glass state is stabilized at 300 K. The glass transition temperature T_g_ becomes equal to 695.5 K instead of 800 K after a cooling rate at 10^12^−10^13^ K/s. The Phase 3 enthalpy coefficient Δε_lg_ = ε_ls_ − ε_gs_ is equal to 0.22335 at low temperatures, and corresponds to a volume change of 0.22335 × 1.5661 = 0.35, in agreement with that of Figure 8.

The ratio T′_g_/T_m_ in Figure 11 is expected to be equal to 1.4467 for T′_g_ = 1818.5 K and T_g_ = 695.5 K and 1.36356 for T′_g_ = 1714 K and T_g_ = 800 K. Its maximum value is 1.4387 at T′_g_ = 1714 K instead of 1.4467. Above T_m_ = 1257 K, the dominant liquid glass phase with T′_g_ = 1818.5 K is progressively replaced by the liquid glass with T′_g_ = 1714 K and T_g_ = 800 K, because ΔV_at_ begins to decrease above 1714 K.

These results show that Δε progressively varies between the two maximum values θ_n+_ = Δε = 0.4467 and 0.36356. The first-order transitions expected at 1714 K and 1818.5 K are not observed because the crystal melting in NiTi_2_ extends up to 2120 K in Figure 3 at 10^13^ K/s. The glass fraction first increases with temperature from Δε = 0.22335 to 0.4387, and attains 0.36356 at 1931 K.

**Figure 12:**
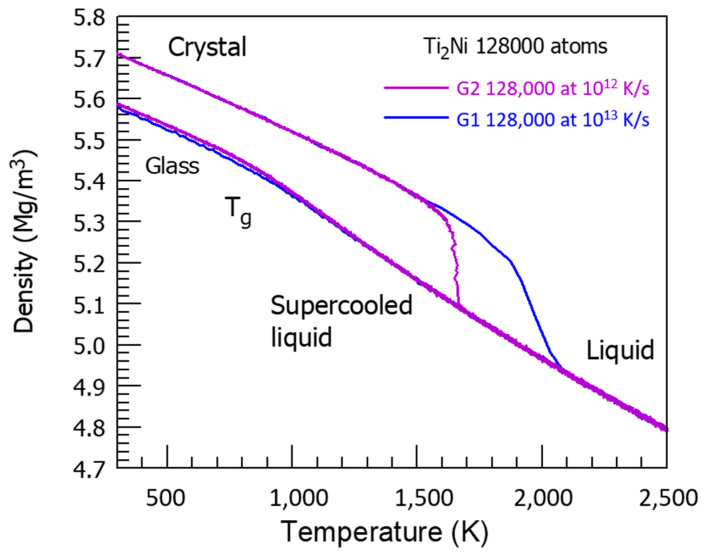
The NiTi_2_ crystal density as a function of temperature at high heating rates. As indicated at 10^13^ K/s, the crystal is completely melted at 2120 K instead of T_0_ = 1257 K at a low heating rate. The glass transition T′_g_ of a glass fraction equal to 0.68632 occurs at θ_n+_ = 0.68632 and T_n+_ = 1.68632 × T_0_ = 2120 K with 0.68632 = 0.14085 + 0.18178 + 0.36369. The crystal melting starts at T_n+_ = 1891 K and θ_n+_ = 0.50454 = 0.14085 + 0.36369. At 10^12^ K/s, melting occurs at 1650 K, corresponding to θ_n+_ = (1650 − 1257)/1257 = 0.31265. The sum of singular coefficients (0.14085 + 0.18178) leads to (0.32263) and to θ_n+_ = 1663 K.

There is a competition between two glass phases. The first one has a glass transition temperature equal to T_n+_ = 2120 K, which is equal to the temperature T_n+_ of NiTi_2_ full melting, as shown in Figure 12, studying the crystal melting temperature dependence with high heating rates at 10^12^−10^13^ K/s. The transition is sharp with 10^12^ K/s, and continuous with 10^13^ K/s.

This temperature T_n+_, being the liquid glass transition temperature, corresponds to θ_n+_ = 0.68632 equal to the sum of singular values (0.14085 + 0.18178 + 0.36369) of the enthalpy coefficient of Phase 3. The NiTi_2_ melting starts earlier at T_n+_ = 1891 K and θ_n+_ = 0.50454 = 0.14085 + 0.36369, and is prolongated toward θ_n+_ = 0.68632. The second glass phase results from a first-order transition obeying to the nucleation law in Equation (7) with a weaker and weaker fraction Δε. The maximum difference Δε increases up to 0.4387 without attaining T′_g_/T_m_ = 0.4467, and declines toward zero at 2120 K. The first-order transition cannot take place at T′_g_ = 1714 K because the glass phase is prolongated by that of a liquid glass fraction equal to 0.686 with T′_g_ = T_n+_ = 2120 K.

The two liquid states found by numerical simulations are already observed in Co–B melts [39]. The temperature-dependent liquid structures are studied in situ, measuring the magnetization. A magnetization anomaly in terms of the non-Curie–Weiss temperature dependence of magnetization was observed in the overheated state, demonstrating a temperature-induced liquid–liquid structure transition. This anomalous behavior was found to be a universal formula for the Co–B binary alloy system. The transition point at T_n+_ (called T_0_ in the publication), above which there is a unique liquid state and below which two paramagnetic Curie temperatures (θ_p_ (L_I_), θ_p_ (L_II_)) corresponding to two distinct kinds of liquids (i.e., high-magnetization liquid (HML)_I_ and low-magnetization liquid (LML)_II_), are measured. With the increased concentration of Co, T_n+_, θ_p_ (L_I_) and θ_p_ (L_II_) shift to higher temperatures, and the Curie constants for the HML and LML decrease. Based on the location of T_n+_, a guideline is drawn above the liquidus in the Co–B phase diagram.

As a conclusion of this chapter, the liquid glass states extend far above T_m_. Table 2, after ref. [26], illustrates the positioning of liquid glasses in terms of connectivity and ordering increase (e.g., on temperature decrease).

The right column of Table 2 is for solid-like matter, which occurs when the degree of connectivity between atomic or molecular constituents is high. Here, we have glasses with a topologically disordered distribution of atoms or molecular species, or crystals. At a medium degree of order, we have composite materials composed of both vitreous, crystalline with periodicity, and even quasi-crystalline (QC) phases with nonperiodic order [29,90]. When the degree of connectivity is low, as shown in the second column of Table 2, we have fluid-like behavior of matter. It starts with melts containing icosahedral superclusters when there is low connectivity between species at a low degree of their ordering. If the degree of ordering becomes high, we have the case of liquid crystals, which is a distinct phase of matter observed between the crystalline (solid) and isotropic (liquid) states. The liquid glass state of matter was missing within classification of matter until recently, because it is a combination of both glassy and liquid fractions, which cannot be readily revealed, e.g., via X-ray diffraction. It is a flowing state of matter placed within a classification scheme at a low degree of connectivity characteristic to liquids, and has a medium degree of ordering because the fraction of glass within liquid adds some degree of ordering, being distinct from the molten state of matter at least by its symmetry signature, i.e., the Hausdorff dimensionality of bonds, which is D = 3 for glasses and D = 2.5 for liquids; see, e.g., Table 2 of Refs. [25,36,91,92,93].

## 5. Conclusions

The formation of liquid glasses induced by first-order transitions at temperatures T_x_ < T_m_, with glass transition temperatures at T_n+_ > T_m_ has been proposed many times during the last years. Glass transitions have been recently observed above T_m_ in the composite (ZIF-62) (Al-rich) (50/50), in tin, and in suspensions of ellipsoidal colloids. The existence of such liquid glasses can be extended by molecular dynamics simulations. NiTi_2_ is chosen because its glass transition, at T_g_ = 800 K (T_m_ = 1257 K) and at high cooling rate, was initially determined by MD simulations, revealing structural changes at this glass transition via radial distribution functions. Applying very high pressures attaining 1000 GPa at 1000 K, and abruptly decreasing the temperature from the liquid state to 300 K or increasing the temperature from 300 K to 1000 K from the glass state, induces a weak reversible first-order transition of the atomic volume with ΔV_at_ = 0.0098 Angström^3^. A new liquid glass state exists at 300 K up to 2120 K after reduction of the pressure to one bar, characterized by an atomic volume difference at 300 K, with the classical glass with T_g_ = 800 K being 35.69 times higher than under a pressure of 1000 GPa. This decrease of ΔV_at_ with pressure is associated with the strong increase of T_m_. [T_m_ (1000 GPa)/T_0_]^2^ is equal to 35.69 because the entropy of melting linearly increases with pressure instead of being constant. The melting temperature T_m_ of NiTi_2_ would attain 6779 K for T_0_ = 1257 K. We control this relation, calculating the melting temperatures of copper and iron as a function of pressure using known experimental values at various low pressures. Our extrapolated values agree with other extrapolations for copper and iron. The observed first-order transitions show that this liquid glass exists up to temperatures T_n+_ = 1.686 T_m_, and that memory effects are present at ambient pressure by abruptly varying the temperature under extreme high pressures. Liquid glasses are a new class of materials which deeply disrupt the concept of homogenous liquids without memory of their solid and glass transformations below T_m_.

The continuous increase of the volume difference ΔV_at_ between the liquid and the glass from 300 K up to T′_g_ = 1714 K shows that the volume fraction involved in this glass fraction attains ~44%.

This finding could lead to a reconsideration of glass formation in volcanic rocks. Up to now, the explanation is based on the high content of SiO_2_ in amorphous rocks with T_g_ ≤ 1473 K. A first-order transition at T′_g_ = 2 T_m_ − T_g_ ≤ 2519 K could exist during cooling or, in the absence of spontaneous first-order transition at T′_g_ ≤ 2519 K, and depending on the thermal history, a first-order transition could be induced during the simultaneous pressure and temperature decreases at different rates.

## Figures and Tables

**Table 1:** Four first columns on the left are devoted to the liquid having T_g_ = 695.5 K, while the four last columns on the right to T_g_ = 800 K. Values of T_m_, Δε, and ΔV_at_ are given as a function of pressure in GPa respecting Equations (16) and (17).

P (GPa)	T_m_	Δε	ΔV_at_ (Å^3^)	P (GPa)	T_m_	Δε	ΔV_at_(Å^3^)
0	1257.0	0.22335	0.34979	0	1257.0	0.18178	0.28469
30	1795.7	0.10945	0.17141	30	1706.2	0.09866	0.15452
60	2206.5	0.07248	0.11352	60	2059.6	0.06771	0.10604
100	2657.3	0.04998	0.07827	100	2452.9	0.04774	0.07476
150	3130.8	0.03600	0.05639	150	2869.7	0.03488	0.05462
250	3909.4	0.02309	0.03616	250	3559.8	0.02267	0.03550
350	4556.8	0.01700	0.02662	350	4136.3	0.01679	0.02629
450	5123.0	0.01345	0.02106	450	4641.7	0.01333	0.02088
500	5383.9	0.01217	0.01907	500	4874.8	0.01209	0.01893
600	5870.9	0.01024	0.01604	600	5310.4	0.01018	0.01595
700	6320.5	0.00883	0.01383	700	5712.9	0.00880	0.01378
800	6740.1	0.00777	0.01217	800	6088.9	0.00775	0.01213
900	7135.2	0.00693	0.01086	900	6442.9	0.00692	0.01084
1000	7509.5	0.00626	0.00980	1000	6778.5	0.00625	0.00979

**Table 2:** Phases of materials as function of connectivity and ordering of atomic constituents.

Degree of Connectivity. *Degree of Ordering*	Low	High
*High*	Liquid crystals; Liquid quasi-crystals	Crystals; Quasi-crystals
*Medium*	Liquid glasses	Glass crystalline materials; Glass quasi-crystalline materials
*Low*	Melts	Glass ^1^

^1^ The connectivity between atomic species can be diminished not only by an increase of temperature: the irradiation of glasses, which breaks the interatomic chemical bonds, leads to fluidization of glasses [89].

## Data Availability

Data supporting reported results can be found within references provided.
